# Utility of population-based HIV impact assessments to understand the associations of stigma with the HIV treatment cascade: Analytical framework using cross-sectional evidence from Tanzania

**DOI:** 10.1371/journal.pone.0323916

**Published:** 2025-05-28

**Authors:** Mohamed F. Jalloh, Alexander Kailembo, Nicolas Schaad, Sophia A. Nur, Prosper Njau, Haruka Maruyama, Kayla Lavilla, Kathy Hageman, Mbaraka Amuri, Nora Hennesy, Eunice Mmari, Mahesh Swaminathan, Leonard Maboko, George S. Mgomella

**Affiliations:** 1 Division of Global HIV and TB, U.S. Centers for Disease Control and Prevention, Tanzania Office, Dar es Salaam, Tanzania; 2 Tanzania Ministry of Health, National AIDS Control Programme, Strategic Information Unit, Dodoma, Tanzania; 3 ICAP at Columbia University, Tanzania Office, Dar es Salaam, Tanzania; 4 U.S. Centers for Disease Control and Prevention, Division of Global HIV and TB, Atlanta, United States of America; 5 Tanzania Commission for AIDS, Dodoma, Tanzania; SKYDA Health Nigeria, NIGERIA

## Abstract

**Background:**

Stigma is a major barrier to ending HIV as a public health threat. We present an analytical framework for quantifying the effects of HIV-related stigma on the treatment cascade using biomarker data from a Population-based HIV Impact Assessment (PHIA) in Tanzania.

**Methods:**

We first reviewed HIV-related stigma items from 15 PHIA surveys in sub-Saharan Africa. Using nationally representative data of 1,831 diagnosed and undiagnosed PLHIV aged 15 and older in Tanzania, we applied modified Poisson regression models to examine associations of stigma with the treatment cascade, adjusting for HIV knowledge and demographics.

**Results:**

We identified 41 unique stigma-related items in 13 of the 15 PHIA surveys. In Tanzania, PLHIV who expressed any stigma driver (stigmatizing attitude, discriminatory attitude, or shame) were 27% less likely to know their HIV status (adjusted prevalence ratio [aPR] 0.73; 95%CI [0.65–0.83], p < 0.001), while those expressing all three were almost never aware of their status (aPR < 0.01; 95%CI [0–0.01], p < 0.001). Stigma drivers were not significantly associated with ART use among diagnosed PLHIV or viral load suppression (VLS) among those on ART. Diagnosed PLHIV who felt the need to hide their status when seeking non-HIV healthcare were 9% less likely to be on ART (aPR 0.91; 95%CI [0.85–0.98], p = 0.013), and those on ART were 10% less likely to achieve VLS (aPR 0.90; 95%CI [0.81–0.99], p = 0.047).

**Conclusions:**

Stigma likely prevented many undiagnosed PLHIV in Tanzania from knowing their status. Fear of healthcare discrimination due to anticipated stigma undermines ART uptake among diagnosed PLHIV and viral suppression among those on ART. PHIA surveys have untapped potential to quantify the effects of HIV-related stigma and inform interventions to end HIV as a public health threat.

## Introduction

Stigma manifests through culture, power, and differences to construct a social order that disadvantages the stigmatized group [1]. In the context of the AIDS pandemic, stigma has been a major barrier to HIV prevention, care, and treatment [2]. People living with HIV (PLHIV) experience different forms of stigma that include labeling, stereotyping, separation, status loss, and discrimination [3]. Stigmatizing experiences may lead to internalized stigma and adverse mental health among PLHIV that can negatively impact their HIV treatment outcomes [4]. Stigma likely hindered achievement of the UNAIDS 90-90-90 targets by 2020 and threatens achievement of the 95-95-95 targets to end the AIDS epidemic by 2030 [5]. These targets aim to get 95% of PLHIV to know their HIV status, 95% of those diagnosed receiving antiretroviral therapy (ART), and 95% of those on ART achieving viral load suppression (VLS) [5]. Eliminating all forms of stigma is an integral component to ending the AIDS pandemic [2,6]. For this reason, in addition to the 95-95-95 targets, the UNAIDS Fast-Track strategy includes an ambitious but important target of achieving zero discrimination, notably through provision of concrete benchmarks and increasing investment in programmatic action to reduce HIV-related discrimination and stigma [5]. Therefore, it is important to evaluate and quantify how stigma impacts the HIV treatment cascade.

A nationally representative study of diagnosed PLHIV in the United States found that stigma was negatively associated with ART adherence, missed HIV care appointments and poor mental health [7]. A longitudinal study among diagnosed PLHIV from South Africa showed that early internalization of stigmatizing attitudes reduced the likelihood of ART initiation and VLS [8]. A cohort study from Zambia and South Africa revealed that diagnosed PLHIV who held stigmatizing attitudes were less likely to initiate ART and less likely to be virally suppressed if on ART [9,10]. Similarly, longitudinal data from Uganda, Kenya, Tanzania, and Nigeria demonstrated a strong negative association of stigma with ART adherence and VLS [11]. However, these prior studies were either not based on nationally representative samples of PLHIV or only comprised cohorts of diagnosed PLHIV who already knew their HIV status.

Behavioral and social research are important to understanding the individual and structural drivers of stigma [12]. There is evidence on effective interventions that can reduce stigma, and in turn, improve HIV treatment outcomes [13–15]. Home-based HIV counseling and testing, for example, has shown promising results in reducing stigma, especially in settings with high HIV prevalence [16]. Addressing the underlying structural elements of stigma that result in discriminatory practices remains a major challenge across diverse settings [17].

Population-based HIV Impact Assessment (PHIAs) have been instrumental in producing biomarker data to measure progress toward the UNAIDS 95-95-95 targets and describe the continuum of HIV services [18]. The PHIA in Tanzania, known as the Tanzania HIV Impact Survey (THIS) 2016–2017, showed that 61% of the approximately 1.5 million PLHIV in the country knew their status, 94% of those who knew their status were receiving ART, and 87% of those on ART were virally suppressed [19]. To our knowledge, the associations between stigma and all three of these outcomes have not been examined comprehensively in Tanzania or elsewhere. To address this gap, we developed and analytical framework to evaluate the associations between HIV-related stigma and biomarker evidence for the HIV treatment cascade outcomes using Tanzania as a case study.

## Methods

In the first part of our study, we reviewed publicly available final reports of PHIAs from the websites of ICAP at Columbia University (https://phia.icap.columbia.edu/), Ciheb at the University of Maryland (http://ciheb.org/Nigeria/), and Human Sciences Research Council (https://hsrc.ac.za/). We reviewed all modules and items in the adult questionnaire in each identified final report, flagged unique HIV-related stigma items, and entered them in Table 1. For each item, we noted the type of response options (e.g., yes/no, agree/disagree, or Likert-type). For each country, we denoted the stigma items included in their PHIA, if any. For each included stigma item per country, we further denoted if the item was asked to all participants (adults and/or adolescents) regardless of their HIV status, asked to only those who self-reported HIV-negative status, asked to only those who self-reported HIV-positive status, or not identified as an item in the publicly available survey questionnaire. A country was excluded from this part of the analysis if the questionnaire for its PHIA survey was not in the publicly available final study report.

**Table 1:** Measures of HIV-related stigmatizing attitudes, health services discrimination, knowledge, treatment cascade, and population viral load suppression among HIV seropositive participants—Tanzania, 2016-2017.

	Item/measure	Classification	Number of Respondents^‡^	% (95%CI)^†^
Number of participants lab confirmed to be HIV-positive (N)			1,831	–
**Stigma**	Would you buy fresh vegetables from a shopkeeper or vendor if you knew that this person had HIV?	Self-reported “no”	1,801	11 (9–13)
	Do you think that children living with HIV should be able to attend school with children who are HIV negative?	Self-reported “no”	1,776	8 (6–9)
	Do you agree or disagree with the following statement? “I would be ashamed if someone in my family had HIV.”	Self-reported “yes”	1,814	10 (8–12)
	Composite: Driver of stigma	Expressing one or more drivers of stigma	1,766	23 (20–26)
	In the last 12 months, when you sought health care in a facility where your HIV status is not known, did you feel you needed to hide your HIV status?	Self-reported “yes”	1,052	2 (1–4)
	In the last 12 months, have you been denied health services including dental care, because of your HIV status?	Self-reported “yes”	1,011	12 (10–15)
**Comprehensive HIV knowledge**	Can the risk of HIV transmission be reduced by having sex with only one uninfected partner who has no other partners?	Self-reported “yes”	1,831	75 (72–78)
	Can a person reduce their risk of getting HIV by using a condom every time they have sex?	Self-reported “yes	1,830	75 (72-78)
	Can a person get HIV from mosquito bites?	Self-reported “no”	1,830	76 (73–78)
	Can a person get HIV by sharing food with someone who has HIV?	Self-reported “no”	1,831	88 (86–89)
	Can a healthy-looking person have HIV?	Self-reported “yes”	1,831	84 (82–86)
**HIV treatment** **cascade**	Awareness of HIV positive status	Self-reported aware or ARVs detected in blood	1,782	61 (57–64)
	Receiving ART among those aware	ARVs detected, self-reported on ART, or both ARVs detected and self-reported ART	1,101	94 (92–95)
	Viral load suppression among ART clients	Viral load test result showed <1000 copies HIV RNA/ mL	1,026	87 (84–89)
**Population viral load suppression**	Viral load suppression among all PLHIV regardless of status awareness of ART use	Viral load test result showed <1000 copies HIV RNA/ mL	1,782	49 (46–52)

ARVs = antiretrovirals; ART = antiretroviral therapy.

‡Total number of de facto household members with a laboratory confirmed HIV-positive status.

†Weighted row percentages and 95% confidence intervals.

In the second part of our study, we retrospectively used Tanzania as an illustrative case study by using cross-sectional data from the THIS 2016–2017, [19] which has been described in detail elsewhere [20,21]. The THIS 2016–2017 was a cross-sectional survey that used multistage cluster sampling to obtain a nationally representative sample of children aged 0–14 years and participants aged 15 years and older. Data collection occurred between October 2016 and August 2017. Our analysis focused on participants 15 years and older who were HIV-positive in the survey regardless of if they knew their status or not at the time of the survey.

HIV serostatus was assessed based on the Tanzania national HIV rapid testing algorithm, [22] starting with a rapid HIV home-based test using SD BiOLINE HIV-1/2 3.0 (Abbott Molecular Inc., Chicago, Illinois, U.S., formerly Alere) and followed by Uni-Gold™ HIV (Trinity Biotech Manufacturing, Ltd., County Wicklow, Ireland). Confirmatory HIV testing was performed using Geenius™ HIV 1/2 Supplemental Assay (Bio-Rad Laboratories, Hercules, CA, U.S.) for all samples that tested positive or had indeterminate test results.

### Measures

Various domains of HIV-related stigma, comprehensive HIV knowledge, sex, age, marital status, education, and urban-rural residence were the explanatory measures in our study. The primary outcome measures were awareness of HIV-positive status among PLHIV, uptake of antiretroviral therapy (ART) among diagnosed PLHIV, and viral load suppression (VLS) among PLHIV on ART (Table 2).

**Table 2:** Prevalence of the drivers of stigma and discrimination disaggregated by comprehensive HIV knowledge and sociodemographic characteristics among HIV seropositive participants, Tanzania, 2016-2017.

	n (%) ^‡^	Expressed any of the drivers of stigma	Hid HIV status when seeking health services	Denied health services due to HIV status
		% (95%CI) ^†^	% (95%CI) ^†^	% (95%CI) ^†^
**Comprehensive HIV knowledge**				
Yes	737 (41)	14 (11-18)	13 (10-18)	2 (1-5)
No	1094 (59)	24 (19-30)	12 (8-19)	2 (1-4)
**Sex**				
Male	564 (34)	24 (19-30)	12 (8-19)	2 (1-4)
Female	1267 (66)	22 (19-25)	12 (10-16)	2 (1-5)
**Age group**				
15-24 years	171 (10)	27 (18-37)	15 (7-29)	6 (1-25)
25-34 years	480 (26)	22 (18-28)	15 (9-22)	2 (1-6)
35-49 years	804 (44)	20 (16-24)	12 (9-16)	2 (1-4)
50-59 years	254 (15)	23 (16-30)	11 (6-19)	0 (0-2)
60 years or older	122 (5)	36 (24-49)	6 (2-18)	2 (0-7)
**Marital status**				
Married	745 (40)	22 (18-27)	14 (11-19)	1 (0-3)
Living together	281 (16)	21 (15-29)	13 (7-21)	5 (2-11)
Widowed	283 (14)	23 (17-29)	7 (4-11)	1 (0-3)
Divorced	167 (9)	18 (13-26)	14 (7-26)	2 (1-7)
Separated	186 (10)	27 (19-36)	10 (5-20)	4 (1-12)
Never married	168 (11)	25 (18-32)	13 (7-25)	3 (0-23)
**Education**				
No formal education	380 (20)	37 (30-46)	9 (5-16)	2 (0-6)
Primary	1263 (68)	20 (17-23)	14 (11-17)	3 (1-5)
Secondary or higher	186 (12)	16 (10-24)	10 (5-18)	^*^
**Residence**				
Rural	1084 (55)	28 (24-31)	12 (9-17)	2 (1-4)
Urban	747 (45)	17 (13-21)	12 (9-16)	2 (1-5)

‡Unweighted frequencies and weighted column percentages.

†Weighted row percentages and 95% confidence intervals.

*No observations.

#### HIV-related stigma.

The THIS 2016–2017 study comprised of five HIV-related stigma items that we used in our study. Three of the items were asked to all PLHIV including those who were undiagnosed at the time of the survey and did not know their HIV status. The final two items were only asked to PLHIV who were already diagnosed prior to the survey.

*HIV-related stigma expressed by any PLHIV (diagnosed and undiagnosed)*

We measured drivers of stigma with three self-reported items from the THIS 2016–2017 that assessed stigmatizing attitude, discriminatory attitude, and shame among all PLHIV, including those who were undiagnosed at the time of the survey and did not know their HIV status (Table 2). Stigmatizing attitude was measured by asking the question: Would you buy fresh vegetables from a shopkeeper or vendor if you knew that this person had HIV? Discriminatory attitude was measured by asking the question: Do you think that children living with HIV should be able to attend school with children who are HIV negative? And finally, shame was measured by asking the question: Do you agree or disagree with the following statement? “I would be ashamed if someone in my family had HIV.” The response option was categorical for each question (yes, no, and refuse to answer). A binary composite variable was then created based on responses from the three items to indicate the expression of one or more (i.e., any) drivers of stigma. A count variable was created for the number of drivers of stigma that participants expressed (zero, one, two, or all three).

*HIV-related stigma anticipated or experienced among diagnosed PLHIV only*

To measure anticipated stigma, diagnosed PLHIV were asked “In the last 12 months, when you sought health care in a facility where your HIV status is not known, did you feel you needed to hide your HIV status?”. To measure experienced stigma, diagnosed PLHIV were asked “In the last 12 months, have you been denied health services including dental care, because of your HIV status?”. The items prompted for ‘yes’ or ‘no’ responses. For each item, we coded the response options ‘1’ to reflect a form of stigma and ‘0’ to reflect absence thereof.

#### Comprehensive HIV knowledge.

We measured comprehensive knowledge based on five self-reported items in the THIS 2016–2017. Each item prompted for ‘yes,’ ‘no’ or ‘don’t’ know’ responses. Two of the items assessed correct knowledge and three assessed misconceptions about HIV. Comprehensive knowledge was coded “1” for respondents who correctly responded to all 5 items and coded “0” for respondents who incorrectly responded to one or more items.

#### Detection of antiretrovirals in blood.

Antiretrovirals (ARV) in blood was assessed using a qualitative assay to detect concentrations of efavirenz, lopinavir, or nevirapine on dried blood spot specimens based on an established liquid chromatography tandem mass spectrometry method [23]. These three ARVs were the most prescribed to ART clients in Tanzania as either first- or second-line regimens at the time of the survey. ARV detection was factored into our calculation of the outcomes for the first and second UNAIDS 90/95 targets. Participants with detected ARVs were considered aware of their HIV status and on ART regardless of what they had self-reported to interviewers.

#### First UNAIDS 90/95: Awareness of HIV-positive status.

Awareness of HIV-positive status and ART uptake were measured based on a combination of self-report and ARV biomarker data. Participants were considered aware of their HIV status if they self-reported their awareness or if ARVs were detected in their blood regardless of their self-reported HIV status. The ARV adjustments were done because some PLHIV respondents may have withheld sharing knowledge of their HIV status from the interviewers although already diagnosed and taking ARVs for treatment.

#### Second UNAIDS 90/95: Uptake of antiretroviral therapy among those aware.

We restricted the measurement of this outcome to the sub-sample of PLHIV who were aware of their HIV status. ART uptake was measured based on a combination of self-report and ARV biomarker data. Participants were considered as receiving ART if ARVs were detected in their blood, they self-reported being on ART, or both.

#### Third UNAIDS 90/95: Viral load suppression.

We restricted the measurement of this outcome to the sub-sample of PLHIV who were on ART. Participants were considered virally suppressed if their HIV viral load test result showed <1000 copies of HIV RNA per milliliter of blood; otherwise, they were considered unsuppressed. Viral load testing was performed with a nucleic acid amplification test for quantifying HIV type 1 using the COBAS® AmpliPrep/COBAS® TaqMan® 48 and 96 (Roche Diagnostics, Indianapolis, IN, U.S.).

### Statistical analysis

For the first part of the analysis, we used Microsoft Excel (Microsoft Corporation, Redmond, Washington) to descriptively analyze stigma-related items in PHIA surveys conducted between 2015 and 2018 from 13 countries in sub-Saharan Africa. PHIA surveys from Lesotho (2016–2017) and South Africa (2017) were excluded from the analysis because their final study reports did not include the survey questionnaire.

In the second part of the analysis, we analyzed THIS 2016–2017 data from Tanzania in Stata version 17 SE (StataCorp LLC, College Station, TX). Using the THIS 2016–2017 data for the national sample of PLHIV, [19] we calculated the proportion of PLHIV who expressed the drivers of stigma. The prevalence estimates were disaggregated by comprehensive HIV knowledge, sex, age group, marital status, education, and rural-urban residence categories. Sampling weights were applied to all proportions, and their 95% confidence intervals (CI) to account for the complex survey design.

We further used the THIS 2016–2017 data to investigate various associations between HIV-related stigma and the treatment cascade. Modified Poisson regression models with robust variance estimation were used to fit seven multivariable models [24,25]. We examined the associations between the drivers of stigma and 1) awareness of HIV positive status among PLHIV, 2) ART uptake among diagnosed PLHIV, and 3) VLS among PLHIV on ART. Furthermore, we investigated the associations of hiding one’s HIV status when seeking health services with 4) ART uptake among diagnosed PLHIV and 5) VLS among PLHIV on ART. Finally, we assessed the association of experiencing denial of health services due to HIV status with 6) ART uptake among diagnosed PLHIV, and 7) VLS among PLHIV on ART. Respondents with missing data were excluded from the models.

**Fig 1:**
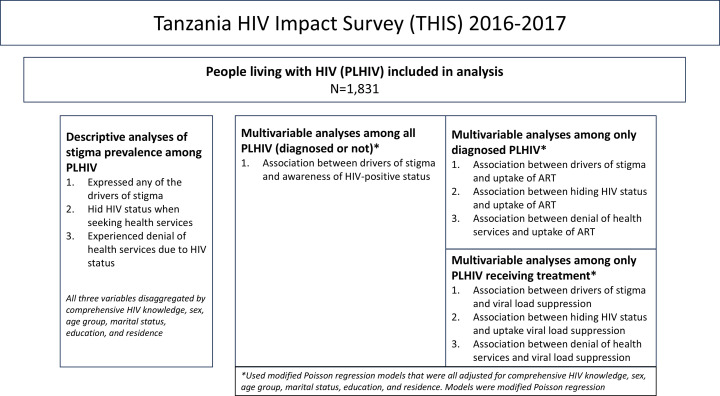
Summary of analysis conducted using data from the Tanzania HIV Impact Survey, 2016-2017.

We adjusted each model for comprehensive HIV knowledge, sex, age, marital status, education, and rural-urban residence categories. Adjusted prevalence ratio (aPR) and corresponding 95% confidence interval were generated for all variables included in each model. A two-sided *P* value < 0·05 was considered statistically significant in all models. The various analyses of the THIS 2016–2017 data are illustrated in Fig 1.

### Ethical considerations

This study was reviewed and approved by the National Institute for Medical Research in Tanzania, Zanzibar Medical Ethics Council, U.S. Centers for Disease Control and Prevention, Columbia University Medical Center, and Westat institutional review boards. Verbal informed consent was obtained in Kiswahili or English from all participants prior to any data collection. Participants 10–17 provided assent to the interview and biomarker components their parents or guardians granted permission to participate in the study. Parents of minors below the age of assent (ages 0–9 years) provided verbal consent for biomarker testing. All consented participants received a hard copy of the same consent form. We accessed the THIS 2016–2017 datasets on June 26, 2022, for the purposes of our secondary analysis. None of the authors had access to information that could identify individual participants during or after data collection.

## Results

### Summary of HIV-related stigma items

**Fig 2:**
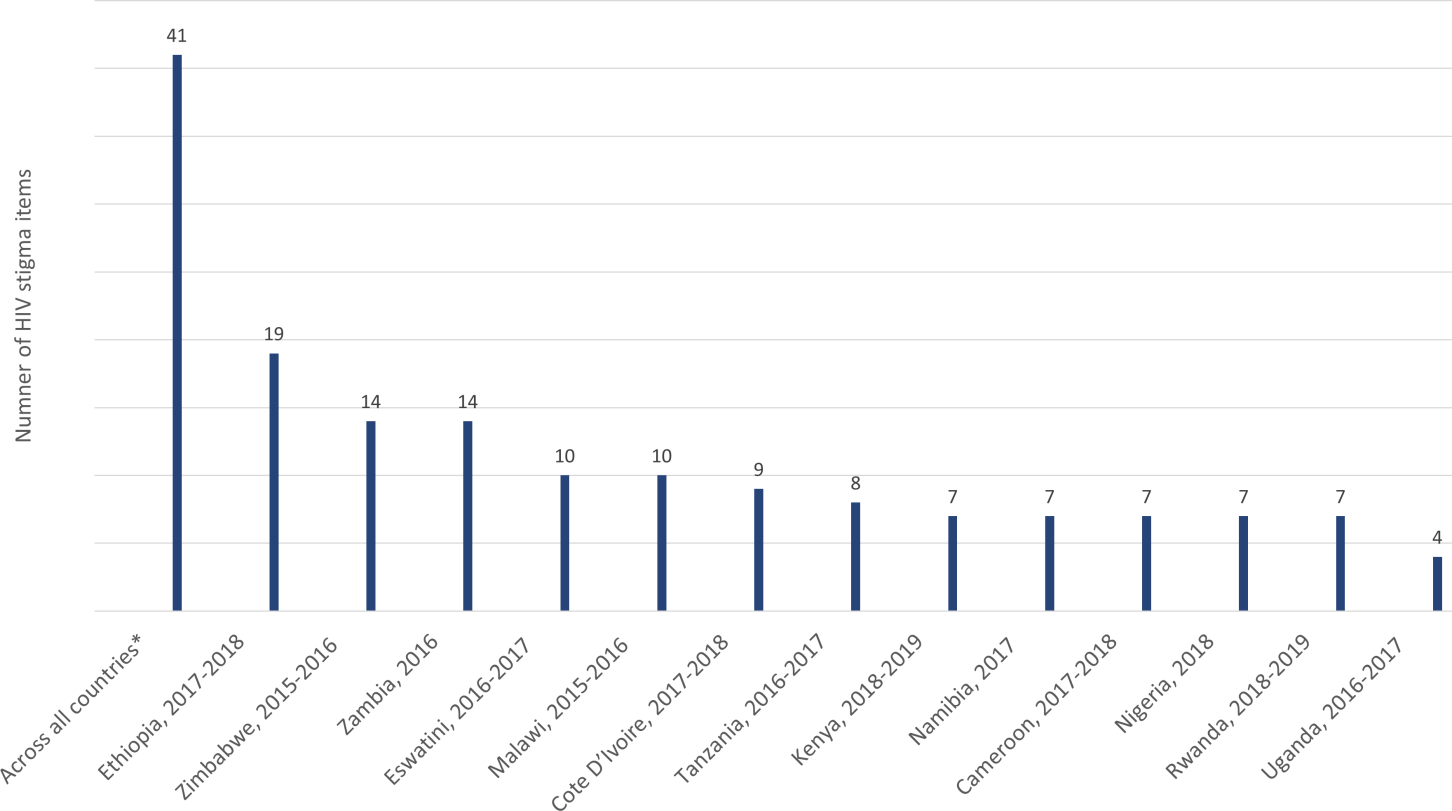
Number of HIV stigma-related items included in Population-based HIV Impact Assessments in countries in sub-Saharan Africa, 2015-2018. Surveys from Lesotho and South Africa conducted between 2015-2018 were excluded because their survey questionnaires were not included in the final study reports.

We identified 41 unique stigma-related items from 13 PHIA surveys that had publicly available questionnaires. The number of items per country ranged from 4 in Uganda to 19 in Ethiopia (Fig 2). All identified stigma items were based on self-reports: 26 had yes/no response options, 6 had agree/disagree response options, 7 had Likert-type scale response options ranging from strongly agree to strongly disagree, and 1 allowed for multiple selections to a list of statements. The two most common questions asked to only self-reported aware PLHIV in 13 (100%) of the surveys were: “In the last 12 months, when you sought health care in a facility where your HIV status is not known, did you feel you needed to hide your HIV status?” and “In the last 12 months, have you been denied health services including dental care, because of your HIV status?” (S1 Table).

### Summary of sample from Tanzania

For our case study in Tanzania, the THIS 2016–2017 sample comprised 1,831 PLHIV, including those who were unaware of their status prior to testing positive for HIV as part of the survey (Table 1). Overall, 1,267 (66%) participants were female, 804 (44%) were between 35 and 49 years old, 745 (40%) were married, 1,263 (68%) had some primary school education, 1,084 (55%) resided in rural areas, and 1,094 (59%) lacked comprehensive HIV knowledge (Table 2).

### Prevalence of HIV-related stigma in Tanzania

Prevalence of any of the drivers of stigma (stigmatizing attitude, discriminatory attitude, or shame) was 23% [95% confidence interval 20%-25%] overall, 37% [29%-46%] among PLHIV with no formal education, and 36% [24%-49%] among PLHIV aged 60 years and older. Among diagnosed PLHIV in our sample, 12% [10%-15%] expressed that they have felt the need to hide their HIV status when seeking health care at a health facility where their status was not known. Denial of health services due to HIV status was experienced by 2% [1%-4%] of diagnosed PLHIV (Table 1).

### Associations between HIV-related drivers of stigma and the HIV treatment cascade in Tanzania

Compared to not expressing any of the three drivers of stigma, PLHIV who expressed at least one of them were 27% less likely to know their status (adjusted prevalence ratio [aPR] 0.73; [95%CI 0.65–0.83], p < 0.001). PLHIV who expressed at least two of them were 33% less likely to know their status (aPR 0.67 [0.55–0.85], p = 0.001). Among all PLHIV, those who expressed all three drivers of stigma were almost never likely to know their status (aPR < 0.01 [0–0.01], p < 0.001). Among PLHIV who knew their status, expressing one or more of the drivers of stigma was not significantly associated with their uptake of ART. Expressing one or more of the drivers of stigma was not significantly associated with VLS among PLHIV on ART (Table 3).

**Table 3:** Association between HIV-related stigmatizing attitudes and the HIV treatment cascade, adjusted for comprehensive HIV knowledge and sociodemographic characteristics—Tanzania, 2016-2017.

	Awareness of HIV-positive status among all PLHIV		Uptake of ART among PLHIV aware of their status		Viral load suppression among PLHIV on ART	
	aPR (95%CI)	*P* value	aPR (95%CI)	*P* value	aPR (95%CI)	*P* value
**Drivers of stigma expressed** ^‡†^						
Zero (n = 1,367; 78.3%)	Reference		Reference		Reference	
One (n = 277; 15.8%)	0.73 (0.65-0.83)	<0.001	1.04 (0.99-1.08)	0.057	0.98 (0.90-1.05)	0.537
Two (n = 91; 5.4%)	0.67 (0.53-0.85)	0.001	1.02 (0.95-1.10)	0.617	0.92 (0.79-1.08)	0.313
Three (n = 12; 0.4%)	<0.01 (0-0.001)	<0.001	–	–	–	–
**Comprehensive HIV knowledge**						
No	Reference		Reference		Reference	
Yes	1.08 (1.01-1.16)	0.024	1.03 (0.99-1.07)	0.963	1.00 (0.95-1.05)	0.912
**Sex**						
Male	0.79 (0.72-0.86)	<0.001	0.95 (0.91-0.99)	0.020	0.94 (0.88-0.99)	0.047
Female	Reference		Reference		Reference	
**Age group**						
15-24 years	0.72 (0.58-0.91)	0.005	0.93 (0.85-1.02)	0.120	0.94 (0.83-1.07)	0.344
25-34 years	0.81 (0.68-0.96)	0.013	0.91 (0.85-0.98)	0.007	0.92 (0.82-1.02)	0.110
35-49 years	1.02 (0.88-1.19)	0.791	0.97 (0.93-1.02)	0.299	0.95 (0.86-1.04)	0.237
50-59 years	1.12 (0.96-1.31)	0.158	1.00 (0.95-1.05)	0.989	1.01 (0.91-1.11)	0.878
60 years or older	Reference		Reference		Reference	
**Marital status**						
Married	Reference		Reference		Reference	
Living together	0.98 (0.88-1.09)	0.728	0.97 (0.91-1.03)	0.251	0.99 (0.92-1.07)	0.767
Widowed	1.08 (0.99-1.19)	0.098	1.03 (0.99-1.06)	0.159	0.94 (0.87-1.01)	0.103
Divorced	0.98 (0.86-1.11)	0.716	0.96 (0.90-1.03)	0.216	0.97 (0.88-1.06)	0.460
Separated	1.01 (0.88-1.14)	0.937	0.97 (0.91-1.04)	0.358	1.04 (0.96-1.11)	0.323
Never married	0.97 (0.84-1.12)	0.684	1.01 (0.96-1.08)	0.630	0.93 (0.83-1.03)	0.165
**Education**						
No formal education	0.93 (0.80-1.07)	0.313	1.02 (0.96-1.10)	0.437	0.97 (0.89-1.07)	0.552
Primary	1.00 (0.89-1.13)	0.963	1.01 (0.95-1.08)	0.732	0.95 (0.88-1.03)	0.217
Secondary or higher	Reference		Reference		Reference	
**Residential setting**						
Urban	Reference		Reference		Reference	
Rural	0.96 (0.89-1.03)	0.250	0.99 (0.96-1.02)	0.465	1.02 (0.97-1.07)	0.483

aPR = adjusted prevalence ratio, ART = antiretroviral therapy, VLS = viral load suppression and PLHIV = People living with HIV.

‡Unweighted frequencies and weighted column percentages.

†Weighted row percentages and 95% confidence intervals.

### Associations between hiding HIV status when seeking health care in facility where status is not known and the HIV treatment cascade in Tanzania

Diagnosed PLHIV who said they needed to hide their status when seeking health care (not necessarily specific to HIV) were 9% less likely to be on ART (aPR 0.91 [0.85–0.98], p = 0.013) compared to those who did not need to hide their status. In addition, PLHIV on ART who felt the need to hide their HIV status when seeking health care were 10% less likely to be virally suppressed (aPR 0.90 [0.81–0.99],

p = 0.047) compared to those who did not need to do so (Table 4).

**Table 4:** Association between experiencing health care discrimination because of HIV status and the HIV treatment cascade, adjusted for comprehensive HIV knowledge and sociodemographic characteristics—Tanzania, 2016-2017.

	Uptake of ART among PLHIV aware of their status		Viral load suppression among PLHIV on ART	
	aPR (95%CI)	*P* value	aPR (95%CI)	*P* value
**Hid HIV status when seeking health services**				
No	Reference		Reference	
Yes	0.91 (0.85-0.98)	0.013	0.90 (0.81-0.99)	0.047
**Comprehensive HIV knowledge**				
No	Reference		Reference	
Yes	1.02 (0.99-1.06)	0.151	1.02 (0.97-1.07)	0.492
**Sex**				
Male	0.94 (0.90-0.99)	0.016	0.94 (0.88-1.00)	0.055
Female	Reference		Reference	
**Age group**				
15-24 years	0.96 (0.89-1.04)	0.300	0.99 (0.87-1.12)	0.838
25-34 years	0.92 (0.86-0.99)	0.014	0.94 (0.85-1.05)	0.301
35-49 years	0.98 (0.93-1.03)	0.501	0.96 (0.87-1.05)	0.364
50-59 years	1.00 (0.96-1.06)	0.849	1.02 (0.92-1.13)	0.688
60 years or older	Reference		Reference	
**Marital status**				
Married	Reference		Reference	
Living together	0.98 (0.93-1.04)	0.535	0.99 (0.92-1.07)	0.884
Widowed	1.03 (1.00-1.06)	0.051	0.94 (0.87-1.02)	0.112
Divorced	0.98 (0.93-1.05)	0.602	0.97 (0.88-1.06)	0.474
Separated	0.97 (0.91-1.04)	0.407	1.02 (0.95-1.10)	0.574
Never married	1.04 (1.00-1.08)	0.062	0.96 (0.86-1.06)	0.372
**Education**				
No formal education	1.02 (0.96-1.09)	0.511	1.02 (0.92-1.12)	0.751
Primary	1.02 (0.96-1.08)	0.599	1.00 (0.91-1.09)	0.933
Secondary or higher	Reference		Reference	
**Residential setting**				
Urban	Reference		Reference	
Rural	0.99 (0.96-1.03)	0.737	1.02 (0.97-1.08)	0.360

aPR = adjusted prevalence ratio, ART = antiretroviral therapy, VLS = viral load suppression and PLHIV = People living with HIV.

### Associations between denial of health services due to HIV status and the HIV treatment cascade in Tanzania

Experiencing denial of health services was rarely reported by 2% [1%-4%] of diagnosed PLHIV and was not significantly associated with ART uptake among diagnosed PLHIV (aPR 0.90 [0.77–1.06], p = 0.200) or VLS among PLHIV on ART (aPR 1.10 [0.99–1.21], p = 0.059) (Table 5).

**Table 5:** Association between experiencing health care discrimination because of HIV status and the HIV treatment cascade, adjusted for comprehensive HIV knowledge and sociodemographic characteristics—Tanzania, 2016-2017.

	Uptake of ART among PLHIV aware of their status		Viral load suppression among PLHIV on ART	
	aPR (95%CI)	*P* value	aPR (95%CI)	*P* value
**Denied health services because of HIV status**				
No	Reference		Reference	
Yes	0.90 (0.77-1.06)	0.200	1.10 (0.99-1.21)	0.059
**Comprehensive HIV knowledge**				
No	Reference		Reference	
Yes	1.03 (0.99-1.07)	0.106	1.01 (0.96-1.06)	0.744
**Sex**				
Male	0.94 (0.89-0.98)	0.007	0.94 (0.88-1.0)	0.052
Female	Reference		Reference	
**Age group**				
15-24 years	0.95 (0.88-1.03)	0.195	0.98 (0.87-1.11)	0.812
25-34 years	0.90 (0.84-0.97)	0.003	0.94 (0.84-1.04)	0.271
35-49 years	0.97 (0.92-1.02)	0.225	0.95 (0.86-1.05)	0.316
50-59 years	0.99 (0.95-1.05)	0.897	1.03 (0.94-1.14)	0.508
60 years or older	Reference		Reference	
**Marital status**				
Married	Reference		Reference	
Living together	0.98 (0.92-1.04)	0.432	0.98 (0.87-1.11)	0.649
Widowed	1.02 (0.99-1.06)	0.204	0.93 (0.86-1.00)	0.064
Divorced	0.97 (0.91-1.04)	0.429	0.96 (0.87-1.04)	0.334
Separated	0.99 (0.93-1.05)	0.757	1.02 (0.95-1.09)	0.627
Never married	1.03 (0.98-1.08)	0.240	0.94 (0.85-1.04)	0.264
**Education**				
No formal education	1.03 (0.96-1.11)	0.340	1.00 (0.91-1.11)	0.925
Primary	1.02 (0.96-1.09)	0.541	0.98 (0.90-1.07)	0.690
Secondary or higher	Reference		Reference	
**Residential setting**				
Urban	Reference		Reference	
Rural	0.97 (0.89-1.06)	0.448	0.93 (0.81-1.07)	0.245

aPR = adjusted prevalence ratio, ART = antiretroviral therapy, VLS = viral load suppression and PLHIV = People living with HIV.

## Discussion

In Tanzania, there was a strong negative dose-response association between expressing HIV-related stigmatizing attitudes, discriminatory attitudes, or shame, and PLHIV’s awareness of their HIV status. PLHIV who expressed all three drivers of stigma were almost never aware of their HIV-positive status compared to those who expressed none. However, we did not find a significant association between these stigma drivers and ART uptake among diagnosed PLHIV or VLS among PLHIV on ART. About one in ten diagnosed PLHIV felt the need to hide their HIV status when accessing health services at facilities where their status was not known. Those who expressed this stigma-driven behavior, due to anticipated stigma, were less likely to be on ART, and among those already on ART, less likely to be virally suppressed. These findings quantify how stigma likely hindered the HIV treatment cascade in Tanzania. The stigma data from PHIAs, when analyzed against biomarker data for the treatment cascade, as demonstrated in our study, hold significant potential for understanding and informing programmatic responses to the effects of stigma on the HIV treatment cascade.

We also found that while having comprehensive HIV knowledge increased the likelihood of being aware of one’s HIV status, the magnitude of the negative association of stigma was significantly stronger than its association with HIV knowledge. Given the cross-sectional design, it is plausible that the HIV diagnosis itself may have helped to improve HIV knowledge due to the counseling received as part of HIV testing services. Male sex was consistently associated with reduced likelihood of all three outcomes, even after adjusting for stigmatizing attitudes and comprehensive HIV knowledge. Younger PLHIV aged 15–24 years and aged 25–34 years were less likely to know their HIV-positive status compared to those 60 years and older, adjusting for stigmatizing attitudes and HIV knowledge. These findings are consistent with other studies demonstrating the need to have targeted interventions for men and young people to close the gap in their awareness of HIV status [26].

The most recent UNAIDS estimates and the THIS 2022–2023 show that the first 95 is the lowest of the three UNAIDS targets in Tanzania [19,27]. Negative associations between stigmatizing attitudes and HIV testing have been established, [28] which may partly explain the negative association between stigma and status awareness among PLHIV in this study. Since the collection of the data used in our study in 2016–2017, Tanzania has made substantial progress in its HIV epidemic response, including closing the gap in status awareness among PLHIV. The THIS 2022–2023 showed substantial improvement in the first-95 outcome such that 83% of all PLHIV in Tanzania knew their HIV status [29] compared to 61% in the THIS 2016–2017 study [19]. Similarly, preliminary findings from the 2021 PLHIV Stigma Index 2.0 in Tanzania suggested a decline in reported stigma experienced by PLHIV in Tanzania [30]. However, the findings were not nationally representative. The suggestive progress made in reducing stigma may have contributed to improving identification of PLHIV in Tanzania, but such a relationship cannot be discerned from the available data. Continued stigma elimination efforts are needed at the individual, interpersonal, organizational, community, and public policy levels to maintain the gains made [17].

Our study reinforces that stigma may still pose a key challenge in the HIV response in Tanzania and may be addressed through targeted and comprehensive interventions. The study’s findings align with existing peer-reviewed literature on the detrimental effects of various forms of stigma on the HIV treatment cascade. Past studies have consistently shown that HIV-related stigma, including internalized stigma and discrimination in healthcare settings, is a major obstacle to timely HIV diagnosis, treatment initiation, and viral suppression [31]. The significant association between stigma and reduced uptake of ART reflects findings from prior studies that call for comprehensive stigma-reduction programs across multiple levels of the social ecology [32,33]. Integrating stigma-reduction efforts into HIV programs, particularly by involving healthcare workers and community leaders, may be critical for improving HIV-related outcomes [33]. The findings from our study suggest that sustained, multi-pronged approaches are necessary to combat stigma and accelerate progress toward the UNAIDS 95-95-95 targets.

The PHIA surveys present a unique opportunity to more comprehensively understand how stigma may be impacting progress toward the UNAIDS 95-95-95 targets by 2030. These population-based surveys offer gold-standard biomarker data that can provide empirical insights on where and how to better target stigma-elimination interventions. The stigma items included in the PHIA questionnaires varied substantially, posing a limitation to comprehensive cross-country analyses. Nevertheless, we identified two items that were consistently captured in all 13 PHIA surveys between 2015 and 2018. The analytical framework based on data from Tanzania can be adapted for further cross-country analyses to better understand the potential effects of stigma on the treatment cascade. Moving forward, using a core set of standardized stigma items in countries implementing PHIAs would better enable future comparative analyses across countries.

We acknowledge several limitations in our study. Firstly, our study did not assess the internalized domain of stigma and lived experiences of discrimination outside of health care settings among PLHIV. Internalized stigma has shown to have a negative association with ART adherence and VLS elsewhere in sub-Saharan Africa among cohorts of PLHIV [9,10]. Another limitation is that key and vulnerable populations may experience unique forms and higher levels of stigma compared to the general population of PLHIV that cannot be ascertained through the PHIA surveys [34]. Finally, the PHIA data are cross-sectional, which means that we cannot infer a causal-effect relationship between stigma and the treatment cascade outcomes. Nevertheless, as additional rounds of PHIA data become available across countries, the analytical framework we provide in our study can be used to produce a more up-to-date understanding of the potential effects of stigma on the HIV treatment cascade as measured by the associations of stigma with outcomes for the UNAIDS 95-95-95 targets.

## Conclusion

Our study showed that the more stigmatizing attitudes PLHIV held, the less likely they were to know their HIV status, with those expressing three stigmatizing attitudes almost never knowing their status. Our findings also suggest that fear of health service discrimination undermines ART uptake among diagnosed PLHIV and viral suppression among PLHIV on ART. Our study indicates that interventions prioritizing stigma elimination may play a crucial role in achieving and maintaining HIV epidemic control. More holistic stigma measurements, including internalized stigma and experiences of discrimination outside healthcare settings, may be needed in PHIAs and similar surveys to enhance the empirical quantification of stigma’s effects on the treatment cascade. The analytical approach of our study can guide future evaluations of HIV-related stigma’s impact on the UNAIDS targets to fast-track ending the AIDS epidemic.

## Supporting information

S1 Table Summary of HIV-related stigma items in 13 Population-based HIV Impact Assessments in sub-Saharan Africa, 2015–2018. (DOCX)
