# Supplementary material for: Utility of population-based HIV impact assessments to understand the associations of stigma with the HIV treatment cascade: Analytical framework using cross-sectional evidence from Tanzania
Source: PLoS One. 2025 May 28;20(5):e0323916. doi: 10.1371/journal.pone.0323916 (PMC12118844; doi:10.1371/journal.pone.0323916)
Supplement: S1 Table — (DOCX) [file pone.0323916.s001.docx]

S1 Table. Summary of HIV-related stigma items in 13 Population-based HIV Impact Assessments in sub-Saharan Africa, 2015-2018

| **Questionnaire item** | **Response Options** | **Cameroon** | **Cote D’Ivoire** | **Eswatini** | **Ethiopia** | **Kenya** | **Malawi** | **Namibia** | **Nigeria** | **Rwanda** | **Tanzania** | **Uganda** | **Zambia** | **Zimbabwe** |
| --- | --- | --- | --- | --- | --- | --- | --- | --- | --- | --- | --- | --- | --- | --- |
| 1. Would you buy fresh vegetables from a shop keeper or vendor if you knew the person had HIV? | Yes/No | NI | AA | NI | NI | AA | AA | NI | NI | NI | AA | NI | AA | AA |
| 1. Do you think children living with HIV should be allowed to attend school with children who do not have HIV? | Yes/No | NI | AA | NI | NI | AA | AA | NI | NI | NI | AA | NI | AA | AA |
| 1. Do you think people hesitate to take an HIV test because they are afraid of how other people will react if the test result is positive for HIV? | Yes/No | NI | AA | NI | NI | NI | AA | NI | NI | NI | AA | NI | AA | AA |
| 1. Do people talk badly about people who are living with HIV, or who are thought to be living with HIV? | Yes/No | NI | AA | NI | NI | NI | AA | NI | NI | NI | NI | NI | AA | AA |
| 1. Do people living with HIV, or thought to be living with HIV, lose the respect of other people? | Yes/No | NI | AA | NI | NI | NI | AA | NI | NI | NI | NI | NI | AA | AA |
| 1. Do you fear that you could get HIV if you come into contact with the saliva of a person living with HIV? | Yes/No | NI | AA | NI | NI | NI | AA | NI | NI | NI | NI | NI | AA | AA |
| 1. Do you agree or disagree with the following statement: I would be ashamed if someone in my family had HIV. | Agree/  Disagree | NI | AA | NI | NI | NI | AA | NI | NI | NI | AA | NI | AA | AA |
|  |  |  |  |  |  |  |  |  |  |  |  |  |  |  |
| 1. Would you be willing to share food with someone who has HIV? | Yes/No | AH-_ | NI | AH- | AH- | NI | NI | AH- | AH- | AH- | NI | AA | AH- | AA |
| 1. Would you be friends with someone who has HIV? | Yes/No | AH- | NI | AH- | AH- | NI | NI | AH- | AH- | AH- | NI | NI | AH- | NI |
| 1. Would you play with someone who has HIV | Yes/No | NI | NI | NI | NI | NI | NI | NI | NI | NI | NI | AA | AH- | AA |
| 1. Would you be comfortable to have a teacher who has HIV? | Yes/No | AH- | NI | AH- | AH- | NI | NI | AH- | AH- | AH- | NI | NI | NI | NI |
|  |  |  |  |  |  |  |  |  |  |  |  |  |  |  |
| 1. If you knew that a shopkeeper or food seller had HIV, would you buy food from them? | Yes/No | NI | NI | NI | NI | NI | NI | NI | NI | NI | NI | NI | NI | NI |
| 1. Would you be willing to care for a family member with AIDS? | Yes/No | NI | NI | NI | NI | NI | NI | NI | NI | NI | NI | NI | NI | NI |
| 1. If a teacher has HIV but is not sick, he or she should be allowed to continue to teach? | Yes/No | NI | NI | NI | NI | NI | NI | NI | NI | NI | NI | NI | NI | NI |
| 1. Is it a waste of money to train or give a promotion to someone with HIV/AIDS? | Yes/No | NI | NI | NI | NI | NI | NI | NI | NI | NI | NI | NI | NI | NI |
| 1. Would you want to keep the HIV-positive status of a family member a secret? | Yes/No | NI | NI | NI | NI | NI | NI | NI | NI | NI | NI | NI | NI | NI |
| 1. Are you comfortable talking to at least one member of your family about HIV/AIDS? | Yes/No | NI | NI | NI | NI | NI | NI | NI | NI | NI | NI | NI | NI | NI |
|  |  |  |  |  |  |  |  |  |  |  |  |  |  |  |
| 1. In the last 12 months, when you sought health care in a facility where your HIV status is not known, did you feel you needed to hide your HIV status? | Yes/No | AH+ | AH+ | AH+ | AH+ | AH+ | AH+ | AH+ | AH+ | AH+ | AH+ | AH+ | AH+ | AH+ |
| 1. In the last 12 months, have you been denied health services including dental care, because of your HIV status? | Yes/No | AH+ | AH+ | AH+ | AH+ | AH+ | AH+ | AH+ | AH+ | AH+ | AH+ | AH+ | AH+ | AH+ |
| 1. In the last 12 months, have health care providers talked badly about you because of your HIV status? | Yes/No | NI | NI | NI | AH+ | NI | AH+ | NI | NI | NI | NI | NI | AH+ | AH+ |
|  |  |  |  |  |  |  |  |  |  |  |  |  |  |  |
| 1. A person with HIV/AIDS should be allowed to work with other people | Strongly agree, agree, disagree, or strongly disagree | NI | NI | AA | NI | NI | NI | NI | NI | NI | NI | NI | NI | NI |
| 1. People who have HIV/AIDS deserve compassion |  | NI | NI | AA | NI | NI | NI | NI | NI | NI | NI | NI | NI | NI |
| 1. People who are suspected of having HIV/AIDS lose respect in the community |  | NI | NI | AA | NI | NI | NI | NI | NI | NI | NI | NI | NI | NI |
| 1. Everyone should get tested for HIV |  | NI | NI | AA | NI | NI | NI | NI | NI | NI | NI | NI | NI | NI |
| 1. Only persons who think they might be infected with HIV should get an HIV test |  | NI | NI | AA | NI | NI | NI | NI | NI | NI | NI | NI | NI | NI |
|  |  |  |  |  |  |  |  |  |  |  |  |  |  |  |
| 1. To what extent do you agree with the following statement: All HIV-negative people should test for HIV every year? Do you strongly agree, agree, disagree, or strongly disagree? | Strongly agree, agree, disagree, or strongly disagree | NI | NI | NI | AA | NI | NI | NI | NI | NI | NI | NI | AA | NI |
| 1. To what extent do you agree with the following statement: Everyone should get tested for HIV. Do you strongly agree, agree, disagree, or strongly disagree? |  | AA | NI | NI | NI | NI | NI | AA | AA | AA | AA | NI | NI | NI |
| 1. To what extent do you agree with the following statement: Only persons who think they might have HIV should get an HIV test. Do you strongly agree, agree, disagree, or strongly disagree? |  | AA | NI | NI | NI | NI | NI | AA | AA | AA | AA | NI | NI | NI |
|  |  |  |  |  |  |  |  |  |  |  |  |  |  |  |
| 1. In the last 12 months, have you been verbally insulted, harassed and/or threatened because of your HIV status? | Yes/No | NI | NI | NI | AH+ | NI | NI | NI | NI | NI | NI | NI | NI | AH+ |
| 1. In the last 12 months, have you been physically assaulted because of your HIV status? | Yes/No | NI | NI | NI | AH+ | NI | NI | NI | NI | NI | NI | NI | NI | NI |
| 1. In the last 12 months, have you lost your job or another source of income because of your HIV status? | Yes/No | NI | NI | NI | AH+ | NI | NI | NI | NI | NI | NI | NI | NI | NI |
| 1. In the last 12 months, have you been dismissed, suspended, or prevented from attending an educational institution because of your HIV status? | Yes/No | NI | NI | NI | AH+ | NI | NI | NI | NI | NI | NI | NI | NI | NI |
| 1. In the last 12 months, has your child/ children been dismissed, suspended or prevented from attending an educational institution because of your or his/her HIV status? | Yes/No | NI | NI | NI | AH+ | NI | NI | NI | NI | NI | NI | NI | NI | AH+ |
| 1. In the last 12 months, have you been forced to change your place of residence or been unable to rent accommodation because of your HIV status? | Yes/No | NI | NI | NI | AH+ | NI | NI | NI | NI | NI | NI | NI | NI | NI |
| 1. Have you tried to get legal redress for any abuse of your rights as a person living with HIV? | Yes/No | NI | NI | NI | AH+ | NI | NI | NI | NI | NI | NI | NI | NI | NI |
| 1. In general, disclosing my HIV status in my community is more likely to lead to negative outcomes. | Agree/ Disagree | NI | NI | NI | AH+ | NI | NI | NI | NI | NI | NI | NI | NI | NI |
| 1. In general, disclosing HIV status to other people in my community can be helpful to me. | Agree/ Disagree | NI | NI | NI | AH+ | AH+ | NI | NI | NI | NI | NI | NI | NI | NI |
| 1. Disclosing HIV status to others can be helpful to me for taking my ARVs regularly. | Agree/ Disagree | NI | NI | NI | AH+ | AH+ | NI | NI | NI | NI | NI | NI | NI | NI |
| 1. I find it easy to disclose my HIV status to other people. | Agree/ Disagree | NI | NI | NI | AH+ | NI | NI | NI | NI | NI | NI | NI | NI | NI |
| 1. I am interested in disclosing my HIV status to more people than I already have. | Agree/ Disagree | NI | NI | NI | AH+ | NI | NI | NI | NI | NI | NI | NI | NI | NI |
|  |  |  |  |  |  |  |  |  |  |  |  |  |  |  |
| 1. In the last 12 months have you experienced any of the following because of your HIV status? Select all responses that apply to you (HEALTH CARE PROVIDER TALKING BADLY ABOUT YOU =A, VERBALLY INSULTED HARASSED OR THREATENED=B, PHYSICALLY ASSAULTED=C, LOST YOUR JOB OR ANOTHER SOURCE OF INCOME,D, DISMISSED OR PREVENTED FROM ATTENDING AN EDUCATIONAL INSTITUTION=E, CHILD BEEN DISMISSED OR PREVENTED FROM ATTENDING AN EDUCATIONAL INSTITUTION=F, BEEN FORCED TO CHANGE PLACE OF, RESIDENCE/UNABLE TO RENT=G, DON’T KNOW= -8, REFUSED= -9 | Multiple selection | NI | NI | NI | NI | AH+ | NI | NI | NI | NI | NI | NI | NI | NI |

AA: Asked to all adults and/or adolescent participants regardless of self-reported HIV status

AH-: Asked to only self-reported HIV-negative participants

AH+: Asked to only self-reported HIV-positive participants (those who did not know their HIV status were not asked these questions)

NI: Not identified in the survey questionnaire
